# Patient Perspectives on Tobacco Use Treatment in Primary Care

**DOI:** 10.5888/pcd12.140408

**Published:** 2015-02-05

**Authors:** Jacqueline R. Halladay, Maihan Vu, Carol Ripley-Moffitt, Sachin K. Gupta, Christine O’Meara, Adam O. Goldstein

**Affiliations:** Author Affiliations: Maihan Vu, Carol Ripley-Moffitt, Adam O. Goldstein, University of North Carolina at Chapel Hill, Chapel Hill, North Carolina; Sachin K. Gupta, Christine O’Meara, Community-Based Family Practice Physicians, Cary, North Carolina. Dr Halladay is also affiliated with the Cecil G. Sheps Center for Health Services Research, Chapel Hill, North Carolina.

## Abstract

**Introduction:**

Evidence-based tobacco cessation interventions increase quit rates, yet most smokers do not use them. Every primary care visit offers the potential to discuss such options, but communication can be tricky for patients and provider alike. We explored smokers’ personal interactions with health care providers to better understand what it is like to be a smoker in an increasingly smoke-free era and the resources needed to support quit attempts and to better define important patient-centered outcomes.

**Methods:**

Three 90-minute focus groups, involving 33 patients from 3 primary care clinics, were conducted. Participants were current or recent (having quit within 6 months) smokers. Topics included tobacco use, quit attempts, and interactions with providers, followed by more pointed questions exploring actions patients want from providers and outcome measures that would be meaningful to patients.

**Results:**

Four themes were identified through inductive coding techniques: 1) the experience of being a tobacco user (inconvenience, shame, isolation, risks, and benefits), 2) the medical encounter (expectations of providers, trust and respect, and positive, targeted messaging), 3) high-value actions (consistent dialogue, the addiction model, point-of-care nicotine patches, educational materials, carbon monoxide monitoring, and infrastructure), and 4) patient-centered outcomes.

**Conclusion:**

Engaged patient-centered smoking cessation counseling requires seeking the patient voice early in the process. Participants desired honest, consistent, and pro-active discussions and actions. Participants also suggested creative patient-centered outcome measures to consider in future research.

## Introduction

That one-half of people who smoke cigarettes will die of a tobacco-related illness, shortening their lives by an average of 10 years (1), is not lost on US tobacco users, most of whom report that they want to quit (2). Nor is it news to health care providers that helping patients live tobacco-free lives is one of the single best ways to reduce disease, disability, and death (3). And although most quit attempts are unsuccessful (2), more than one-half of people who have tried to quit are no longer smoking, suggesting that those who keep trying can eventually succeed.

Decades of intervention research indicate that evidence-based assistance, including pharmacotherapy and counseling, significantly improves success rates (4,5). Receiving even brief advice from a physician increases quit rates, compared with no advice or usual care, and intensive advice is better than minimal advice (4). Quitlines, available in all 50 states, provide an important self-management support resource for smokers (6). Still, only one-third of cigarette users who try to quit use any assistance (7). A critical challenge, then, is to increase the use of evidence-based treatment of tobacco dependence.

Every primary care visit offers the potential to ensure that tobacco users are aware of the resources available to them. However, counseling for quitting occurs in only 20% to 50% of physician visits and 10% to 24% of dental visits (8–10), and cessation medications are ordered in less than 8% of visits (2,8). Despite waves of antismoking educational initiatives and ongoing efforts to encourage patients to ask questions during routine care, patients cannot necessarily be relied upon to initiate discussions with their providers.

To better understand and address how patients who continue to smoke view or use available cessation resources, it is essential that patient opinions and ideas guide the research process. The objective of this research was to conduct an in-depth exploration of smokers’ personal experiences and ideas about smoking and smoking cessation. The outcomes may help clinicians better understand the resources needed to support patients’ quit attempts and to identify important patient-centered outcomes.

## Methods

### Participant recruitment

Eligible participants were English speakers aged 18 or older who reported smoking or quitting in the previous 6 months and attended 1 of 3 primary care practices in a network of medical practices located in 9 counties in North Carolina. The study took place from September 2013 through September 2014. Recruitment flyers were placed in waiting and patient rooms. In 2 practices, letters were sent to patients whose medical record indicated current smoking. Of the 47 patients who expressed interest, 33 could attend the session times that worked for the majority. Each participant received $50 for participating in one focus group. The University of North Carolina Biomedical Institutional Review Board approved this study.

### Focus groups

Focus groups were held at a practice location or wellness center. The sessions were conducted by 3 research staff members trained in qualitative methods. A series of questions about tobacco use, quit attempts, and interactions with providers was followed by more pointed questions exploring opinions on 1) existing evidenced-based practice and community-level supports (5), 2) relevant patient-centered outcome measures, and 3) interventions that may enhance success in reducing or eliminating tobacco use. Because active solicitation of patient views about choices of outcomes in research is limited (11), we asked participants to suggest outcome measures that could be explored or enhanced in future cessation research.

### Analysis

Focus groups were audiotaped and transcribed verbatim and imported into ATLAS.ti 6.2 (Scientific Software Development GmbH). A codebook of operational definitions was created using the interview guide questions. Inductive coding techniques as described by Strauss and Corbin (12) were used along with the constant comparison method (13). Two investigators met to reach consensus on codes and set rules for salient themes and then independently coded each transcript. Discrepancies were resolved through discussions with study team members. We identified prominent themes within and across practices. We selected quotes that best represented the themes and created 3 acronyms to link participant quotes to their practices (primary care practice [PCP]1, PCP2, and PCP3).

## Results

Most participants were female, were middle-aged, and smoked daily (Table 1). All had health care insurance, and 67% made a quit attempt in the previous year. Seventy-nine percent had been offered quit assistance by a provider. The 3 groups were similar in demographic characteristics and behavior in the group interviews. Participants treated one other with concern and respect and shared fairly equally in responding to questions.

**Table 1:** Characteristics of 33 Focus Group Participants From 3 Primary Care Practices, North Carolina, 2013–2014

Characteristic	Value^a^
**Age, mean (range), y**	53 (20–77)
**Sex**	
Male	30
Female	70
**Cigarette use**	
Smoke every day^b^	73
Smoke some days^c^	6
Recently quit smoking	21
**Other**	
Have medical insurance	100
Made a serious attempt to quit smoking in the past year	67
Has had a doctor offer guidance to quit smoking	79

a All values are percentages unless otherwise indicated.

b Mean number of cigarettes smoked per day, 16 (range, 4–40).

c Mean number of cigarettes smoked per day, 6 (range, 2–10).

Four themes (and multiple subthemes) emerged: 1) the experience of being a smoker, 2) the medical encounter, 3) high-value actions for practices and communities, and 4) new or enhanced patient-centered metrics. The themes were derived inductively or arose directly from discussion questions. Overall thematic content was generally distributed equally among the practice focus groups. 

### Theme 1: The experience of being a smoker 

Personal, social, health risks and financial issues were linked with smoking and the pleasure experienced.

#### Inconvenience

Hidden costs and the near-constant mental acrobatics of planning where and when one can smoke and how to cover it up were a common complaint. “There are so many things you do and . . . so many more costs to it because you’re buying candles and sprays and soaps and creams, and it’s . . . crazy” (PCP1). “You can’t just go stand outside of a building and smoke anymore, so we are always sneaking around and figuring out . . . if I go to this event, where can I go to smoke?”(PCP3). “I think that’s been my most difficult challenge with quitting . . . as soon as you wake up the ritual has begun. Before you go to bed, the ritual has begun” (PCP2).

#### Shame

Shame is evidenced by the lengths people go to hide the fact that they smoke and by the negative self-images they adopt. “No one else I work with smokes, so I’m always the stinky person” (PCP2). “I’m always hiding it somewhere trying to have a cigarette or sneaking somewhere” (PCP2).

#### Isolation

Today’s smokers feel that they are unwelcome and that they miss out because of smoking. “I leave a conversation because I feel like it’s time for a cigarette and you come back and you’re not part of it anymore” (PCP3). “Society has placed such a stigma on it. . . . You almost have to move to France to be accepted as a smoking person” (PCP2).

#### Risks

They are aware of their risks and are conflicted about the consequences of quitting or not. “It’s either gain weight or be in the wooden coffin . . . it’s terrible” (PCP1).

#### Enjoyment

Despite the drawbacks related to the previous subthemes, quitting means giving up something pleasurable and for some, an activity they enjoy most. “Honestly . . . I very much so purely love smoking” (PCP2). “The first thing I want to go for in the morning . . . cup of coffee and a cigarette” (PCP2). 

### Theme 2: The medical encounter

Participants freely discussed past encounters and ideal elements of a helpful medical visit.

#### Expectations

Participants believe most smokers want to quit and expect providers to address smoking at every visit. **“**Smoking’s important . . . it should be every time a person comes in . . . [like when they] take my blood pressure” (PCP1). “Nobody that smokes for 10 years wants to smoke. Okay, it was fun when you’re a kid, but after 10 years . . . you don’t want to do it. You don’t want to spend the money [and time] on it” (PCP1). 

#### Trust

Participants said that providers are in uniquely trusted positions. “The only person who is in the position to help me is that doctor because I trust him. I’m not going to let somebody help me get rid of my addiction that takes up 2 hours a day that I’ve been doing for over 30 years that I don’t trust” (PCP1).

#### Respect

Each encounter must be conducted with respect. Disdain or disrespect is readily detected, promoting anger and a reluctance to share accurate information. “[A provider should not] “shake her finger at me . . . or tell me all of the terrible things that are going to happen to me . . . it’s almost intellectually offensive for a doctor to tell you that smoking is bad for you . . . we know it’s bad for us” (PCP2). “They don’t tell you anything that you haven’t heard a million times. You end up getting fussed at. Then that makes you want to lie. I’ll . . . say I don’t smoke, or if I smoke 10, I’ll say I smoke 5” (PCP1).

A common frustration with patient surveys is that although participants take time to fill them out, clinicians do not acknowledge the effort. “[You] sit in the waiting room . . . fill out 15 minutes worth of paper work. You go in . . . and they’ll ask you the exact same questions. Did you read it? No, they didn’t” (PCP1).

#### Positive and targeted messaging

Participants advocated for positively framed verbal and written messages, ideally targeted to individual circumstances, using language such as “Listen, just say the word, we have plenty of things for you to help and just let me know; this is what works” (PCP2). “When [you] quit smoking, here’s what improves” (PCP2). One participant chose to quit because the information was presented in the context of her overall heart disease risk: “I’m on statins for cholesterol, and I’ve been getting a lot of joint pain. . . . I said, ‘Look, I am done with these statins.’ . . . And she said, ‘I absolutely can’t take you off the statins because as a smoker your risk of a heart attack is like 34% more” (PCP2).

### Theme 3: High-value actions for practices and communities

Participants had several suggestions.

#### More dialogue

More direct and more frequent verbal discussions are needed with providers and staff on the risks and benefits of each treatment option.

Society needs more connection with people . . . I don’t want to check yes or no. I want you to ask me my true need and to give me something to help it and then I want you to follow up. . . . Have one of your nurses call “Hey, how’s that working or how are you feeling?” ’Cause there’s many times I’ve gone home . . . on some medication and it was not right [PCP1].

#### Using the addiction model

Participants welcomed the treatment of smoking as a serious addiction. Several noted that it was easier to stop using narcotics and alcohol than nicotine. They wanted comparable supports, even inpatient services. “It’s just like drug addiction . . . [it] has to be a part of the disease model, and they have to accept it as such” (PCP2). “Like AA, where there are the tobacco users that successfully quit that we can team up with to go to a meeting and hear positive things about having quit themselves” (PCP1). “Why don’t they have Nic-anon?”(PCP2). “They need to make a little house that you can check into for 30 days” (PCP2).

#### Point of care nicotine replacement therapy

Participants were interested in over-the-counter nicotine replacement therapy (NRT), but they were concerned about perceived costs and safe usage and lacked confidence in succeeding alone. “What does this patch do? What can I do on this patch? How is this going to affect me?” (PCP2). “Ninety dollars! What if it doesn’t work?” (PCP2).

Participants suggested that patients have a sample nicotine patch placed and have their questions answered at the same time during a visit. It was suggested that by experiencing immediate relief from nicotine withdrawal, patients may be more empowered to commit to cessation plans. “Hand me a patch. You can even put it on my arm while I’m sitting on the table” (PCP1). “I think it’s a great idea to give something on the way out. . . . It gives me a choice, and smokers have so little choice. . . . If they handed you something that you could try, and if it cut that craving right then and there . . . you’re going to call that doctor back” (PCP1).

#### Educational materials

Several participants pointed out that materials should be available for review before they see their provider, so they can better prepare for their provider encounter. “A lot of times when doctors give you things, it ends up in the car seat and I never read it. If they give it to you when you walk in the examination room, you’d literally have something to read while you’re sitting there” (PCP1).

#### Carbon monoxide monitoring

Participants suggested that carbon monoxide monitoring is a powerful motivator, especially for those without obvious smoking-related symptoms or those persuaded by quantitative data. “Here’s physical proof that you’re doing the right thing for your body” (PCP2). “It’s that affirmation model. It’s something that’s giving you that positive feedback. . . . You met that goal . . . I’m proud of you”(PCP2).

#### Comprehensive infrastructure needs

When time and office capacity are not sufficient for providing a comprehensive bundle of services, participants expect to be referred to quitlines or other counseling resources. “I think, this is probably far-fetched, but I think every physician should have somebody like a tobacco cessation counselor . . . and have [the counselor] call me” (PCP1). “If the [provider] doesn’t have the time or doesn’t have the resources, then I, personally, prefer not even to bring it up” (PCP 1). “Put me on a list [and have] somebody call me” (PCP1).

Moreover, discussions should not be limited to primary care practice appointments: “Every health care provider, every doctor, every neurologist, every dentist, everyone who does anything in health care” (PCP1).

### Theme 4: New or enhanced patient-centered metrics

Participants suggested many types of measurable outcomes. The suggestions were grouped into 3 main categories: decreased use of tobacco, quality of life and wellness, and the patient–physician interaction (Table 2). 

**Table 2:** Outcome Measures for Future Projects and Studies Suggested by Focus Group Members, North Carolina, 2013–2014

Measures of patient behavior change
Decreased use of tobacco (cutting back, not necessarily quitting)
Changes in ratio of nicotine replacement therapy to number of cigarettes smoked
Use of educational materials
Use of support (eg, quitlines, group, family and friends)
**Quality of life and wellness**
Ability to be active without feeling short of breath
Increased sense of taste and smell
Improved dental health
Increased energy level
Reduced sense of isolation and stigmatization
Increased available time to focus on other activities
Sense of freedom in no longer needing to arrange schedule to accommodate tobacco use
Sense of pride in setting example for others or pride showing others that they can reach goals
**The patient–provider interaction**
Patient feels respected
Patient feels encouraged and supported
Patient does not feel judged and is empowered to attempt to make quit attempts
Patient feels that the provider understands how difficult it is for patients to quit
Patient feels that the provider or intervention helped in connecting with unique and personally derived reasons to quit

## Discussion

We conducted a comprehensive, qualitative exploration of the patient perspective on smoking cessation among 33 primary care patients from 3 primary care clinics in North Carolina. Participants offered poignant examples of unhelpful experiences and provided a smoker’s perspective on interventions and outcomes metrics. The most noteworthy suggestions were using positive messaging, making sure patients have ample time to review tobacco-related information before provider encounters, enhancing confidence in the use of NRT by starting it during office visits, embedding assistance in an addiction model, having more frequent verbal communications with clinical staff, incorporating carbon monoxide monitoring as a tool, and connecting smokers with additional resources. The effectiveness of most of these suggestions is supported in the literature (4,14–18), but others merit further study and inclusion in new or enhanced strategies for engaging patients.

Directly engaging patients in the design of office-based smoking cessation interventions has received little attention in the past decade. However, a few studies, focused on subgroups, support similar patient experiences and suggestions. One study used a series of focus groups and an expert panel to help design a smoking cessation program for a Veterans Affairs women’s clinic (19). These patients sought choice through various quit options, with a particular preference for medications, telephone follow-up, and women-only group sessions. Another study surveyed 375 tobacco users that visited emergency departments in 10 urban medical centers across the United States about intervention preferences (20). The study found that tobacco users preferred a range of services to support quit attempts, especially medications, followed by telephone-based counseling and one-on-one counseling. Another study assessed the social, cultural, and educational barriers to smoking cessation services in HIV-positive individuals (21). Here patients wanted more targeted information on the effects of smoking, the difficulty of quitting, and the interactions of cessation and HIV medications.

Our participants made practical, patient-centered recommendations that are consistent with evidenced-based guidelines on behavioral and pharmacologic therapies and that can be implemented with minimal burden on a practice. These include ensuring that providers 1) show more awareness of the increasing isolation that smokers’ experience, 2) provide attractive and positively framed materials while patients are waiting to be seen, 3) address smoking at every visit, and 4) acknowledge and help patients deal with issues of addiction in written and spoken communications. Some patient-generated recommendations and outcome measures could be tested in future effectiveness studies or quality-improvement initiatives, such as combining carbon monoxide monitoring with the placement of NRT patches at the point of care in conjunction with more authentic outreach to patients between visits.

This research has several limitations. Although participants were recruited from general primary care clinics, all participants had insurance and the regional smoking rate was lower than the national rate (22). Our sample was 70% women; men may prefer different approaches. A study of emergency department patients found that male sex and less education were positively related to greater receptivity to smoking cessation counseling (20). When we asked if different resources were necessary according to sex, age, or other subgroups, we heard repeatedly that such differences were irrelevant because all smokers are united in being smokers — the critical factor when considering needs and challenges.

Participants in our study may also have been particularly motivated (21% had recently quit, 67% had made a serious attempt to quit, and 79% reported receiving provider guidance to quit). People less interested in quitting may need approaches other than those identified by our sample. However, in a study published in 2012, 68.8% of US adult smokers reported that they want to quit completely, and 42.7% went at least 1 day in the previous year without a cigarette in an attempt to quit (8). These data suggest that the level of motivation in our sample may approach the norm.

True patient-centered research exists when the patient voice is sought early in the planning process. In this study, we obtained insight into various issues centering on the experience of being a smoker, quitting, and interacting with health care systems and providers. Because of the high prevalence of smoking in their patient populations and the commitment of the primary care workforce to prevent the development and progression of chronic diseases, practice-based research networks, such as those supported by the Agency for Healthcare Research and Quality, are particularly well suited to further develop ideas expressed by our participants to augment smoking cessation interventions. We hope our work encourages others to engage in testing new patient-centered interventions, outcomes, and dissemination strategies to help smokers achieve tobacco-free lives.
